# Optimization of L-PBF Process Parameters for Defect Reduction and Mechanical Strength of Ni-Cr-Mo-Nb Superalloy Using Multi-Objective Methods

**DOI:** 10.3390/ma18081743

**Published:** 2025-04-10

**Authors:** Anton V. Agapovichev, Alexander I. Khaimovich, Vitaliy G. Smelov, Viktoriya V. Kokareva, Vyacheslav P. Alekseev, Evgeny V. Zemlyakov, Anton Y. Kovchik

**Affiliations:** 1 Engine Production Technology Department, Samara National Research University, 34 Moskovskoye Shosse, 443086 Samara, Russia; berill_samara@bk.ru (A.I.K.); smelov@ssau.ru (V.G.S.); kokareva.vv@ssau.ru (V.V.K.); alekseev.vp@ssau.ru (V.P.A.); 2 Turbomachinery and Heat Transfer Laboratory, Aerospace Engineering Department, Technion-Israel Institute of Technology, Haifa 3200003, Israel; 3 World-Class Research Center “Advanced Digital Technologies”, State Marine Technical University, 190121 Saint Petersburg, Russia; e.zemlyakov@ilwt.smtu.ru (E.V.Z.); akovchik@yandex.ru (A.Y.K.)

**Keywords:** laser powder bed fusion, Ni-Cr-Mo-Nb-based superalloy, printability window, grey relational analysis, response surface methodology, gradient ascent method, process parameters, multi-objective optimization

## Abstract

In the context of Additive Manufacturing (AM), particularly the Laser Powder Bed Fusion (L-PBF) technique, optimizing process parameters is essential for achieving dense, defect-free materials. This study investigates the optimization of L-PBF process parameters for a Ni-Cr-Mo-Nb-based superalloy using an integrated three-stage methodology. Stage A applies Grey Relational Analysis to identify the most favourable parameter sets. Stage B uses Response Surface Methodology to develop regression models that correlate process parameters with material characteristics, introducing the specific energy of layer fusion as a key factor. Stage C employs the Gradient Ascent Method to determine the global optimum using a desirability function. The proposed approach reduces the number of required experiments while ensuring optimal mechanical properties: yield strength of 774.73 ± 4.94 MPa, tensile strength of 1022.83 ± 5.19 MPa, and elongation at break of 23.1 ± 0.70%, with minimal LoF area (0.003 mm^2^) and gas pore diameter (0.02 mm). The results demonstrate that integrating Grey Relational Analysis, Response Surface Methodology, and the Gradient Ascent Method effectively identifies the printability window, accelerating material characterization.

## 1. Introduction

The modern pace of development in the aviation and aerospace industries requires the implementation of manufacturing technologies that enable rapid, cost-effective production of high-quality components with minimal post-processing. The experience of companies such as Relativity Space, SpaceX, Rocket Lab, GE Aviation, Rolls-Royce, Safran Aircraft Engines, Pratt and Whitney, and others has demonstrated that the use of additive manufacturing technologies significantly reduces the time required for the technological preparation of complex products. These technologies allow the introduction of fundamentally new design and technological solutions, reduce labour intensity and production costs, and improve the environmental efficiency of the products [1,2,3,4,5,6,7,8].

Laser powder bed fusion, also known under the trademarks Selective Laser Melting or Direct Metal Laser Sintering, is a category of additive manufacturing that uses high-power lasers to create three-dimensional physical objects by melting metallic powders [9]. The quality of parts produced using the L-PBF technology is influenced by a large number of process parameters [10]. By correctly understanding and managing these parameters, it is possible to produce parts whose quality is comparable to those manufactured using traditional methods. In particular, laser parameters such as power (*P,* W), scanning speed (*V*, mm/s), hatch spacing (*h*, mm), and layer thickness (*t*, mm) play a crucial role in determining the density and mechanical properties of the parts [11]. The aforementioned process parameters are commonly combined into a single indicator—the volume energy density (*E_V_*, J/mm^3^), which is defined by Equation (1) [12].

(1) $$E_{V}=\frac{P}{V\cdot h\cdot t}$$

Many researchers have established that the number of microstructural defects, such as gas pores and LoF pores, directly affects the mechanical properties of parts produced using the L-PBF technology [10,13]. Completely eliminating porosity in materials manufactured by L-PBF remains a challenging task, even with optimized process parameters and advanced technologies [13,14]. Even if porosity is significantly minimized through optimized L-PBF parameters [15], this does not guarantee achieving the desired microstructure, strength, and ductility simultaneously [13]. Additionally, minimizing porosity often comes at the cost of reduced production speed [13,16].

The quality of material produced using the L-PBF method is determined not only by its level of porosity but also by its mechanical properties. In addition to minimizing porosity, it is essential to achieve maximum values of mechanical properties such as yield strength (*σ*_0.2_), ultimate tensile strength (*σ_v_*), and elongation at break (δ). These properties are key indicators of reliability and are taken into account when designing components.

However, optimizing the structure and mechanical properties of the material often requires a trade-off between their optimal states. This is due to the varying importance of material characteristics depending on the specific requirements of the product. Moreover, the mechanical properties of the material and the number of microstructural defects may exhibit either weak or even negative correlations [17].

In many studies, L-PBF process parameters have been optimized for a single characteristic, such as minimizing porosity, maximizing mechanical properties, or optimizing the microstructure [17,18,19,20,21]. However, very few works are dedicated to the simultaneous optimization of multiple material characteristics, which represents an important challenge.

Beyond process parameters, another critical factor influencing mechanical properties and microstructural evolution is sample geometry. In several studies, the role of part geometry has been examined, particularly for Ti-6Al-4V [22,23]. These works demonstrate that thermal cycling effects due to geometry variations can significantly impact phase distribution and mechanical performance. Therefore, we introduced an additional research factor—sample thickness (*S*)—to analyze the influence of geometry on the mechanical properties and microstructure of Ni-Cr-Mo-Nb alloys.

Experimental design plays a key role in understanding complex systems, identifying cause-and-effect relationships, and making informed decisions. Statistical analysis and design of experiments (DoE) are widely used to optimize L-PBF process parameters. DoE involves several assumptions, including independence, normality, and homogeneity of variance. In some cases, a linear relationship between variables and the response is assumed in the response surface methodology (RSM) [20,24,25].

In cases where the use of DoE results for the purpose of optimizing process parameters is ambiguous, Grey Relational Analysis (GRA) can be applied [26]. In grey relational analysis, the dimensions of the considered factors are usually different, and their magnitude difference is large. Therefore, the original data are normalized to bring the magnitude of the original data to the order of one and make it dimensionless [27]. This optimization method has been previously described by us in detail [28].

The objective of this study is to develop an optimized methodology for process parameter selection in L-PBF to simultaneously enhance mechanical properties and minimize microstructural defects in Ni-Cr-Mo-Nb alloys. To achieve this, a three-stage optimization framework is introduced, integrating Grey Relational Analysis, Response Surface Methodology, and the Gradient Ascent Method. Additionally, the study investigates the role of sample geometry, specifically sample thickness (*S*), in influencing material properties. The proposed approach aims to provide a more comprehensive understanding of the relationships between process parameters, material microstructure, and mechanical performance, ultimately contributing to improved reliability and efficiency in additive manufacturing of Ni-based superalloys.

In this study, the optimization process is divided into three stages to ensure a comprehensive search for the best process parameters:

Stage A: The GRA method is applied to identify two process parameter sets that exhibit the highest values of grey relation coefficients. These sets are considered the most preferable due to their optimal combination of material characteristics.

Stage B: Based on the GRA results, additional L-PBF experiments are conducted within the factor ranges of the two selected parameter sets. The goal is to derive regression dependencies of material characteristics using the RSM. It is assumed that the global optimum of all characteristics lies within the vicinity of these two selected parameter sets.

Stage C: The global optimum of the combined factors is determined using the gradient ascent method. This conventional optimization approach involves constructing a desirability function that assigns a “score vector” to a set of responses and selecting factor settings that maximize that score. The method is widely used to ensure convergence and stability in optimization problems.

## 2. Materials and Methods

### 2.1. Experimental Design and Sample Preparation

The initial experiment, corresponding to Stage A, was conducted using a combined D-optimal design 4^4^ × 2^2^, transformed from 4^5^//D16.

As the material characteristics under investigation (experiment responses), we considered the following: yield strength (*σ*_0.2_), ultimate tensile strength (*σ_v_*), elongation at break (*δ*), microstructure desirability rank (*Rank*), the area of Lack of Fusion (*LoF*), and the diameter of gas pores (*d*).

The following process parameters were selected as factors for the study: laser power (*P*), volume energy density (*E_V_*), scanning speed (*V*), hatch distance (*h*), and layer thickness (*t*). Table 1 presents the experimental design, where the investigated process parameters are represented within relatively wide ranges. Each experiment was repeated three times. In addition, sample thickness (*S*) was considered as an additional factor. The preheating temperature was set at 80 °C, and a stripe scanning strategy with a 67° rotation was applied. The L-PBF process was carried out in a protective argon atmosphere with a minimum purity of 99.999%. The argon flow rate was maintained at approximately 0.6 m^3^/h during the building process, with an initial consumption of up to 3 m^3^. The chamber pressure was kept between 5 and 5.5 bar.

The specimens were manufactured using an SLM 280HL 3D printer (SLM Solutions Group AG, Lübeck, Germany) equipped with a laser with a maximum power of 400 W. A metallic powder of a Ni-Cr-Mo-Nb-based superalloy was used as the feedstock material.

### 2.2. Microstructural Characterization and Mechanical Property Tests

A scanning electron microscope (Tescan Vega, Brno, Czech Republic) equipped with an energy-dispersive X-ray spectrometer (INCAx-act, Oxfordshire, UK) was used to study the powder surface morphology and chemical composition. This method allowed for a detailed examination of the powder’s structure and properties, which is a crucial step in assessing the quality of the feedstock material.

Microanalysis was performed using a METAM LV-41 optical microscope (LOMO, St. Petersburg, Russia) in a bright-field mode at magnifications of ×50 to ×200. The obtained microstructure images were processed using the specialized software SIAMS (SIAMS Ltd., St. Petersburg, Russia).

Sample etching was carried out using the electrolytic method at room temperature for 5 to 10 s in an electrolyte with the following composition: 10 g of citric acid + 10 g of ammonium chloride + 1 L of water.

To establish dependencies and correlations between printing parameters and the material’s microstructure, the microstructure desirability rank was determined for each experiment based on the criteria presented in Table 2.

Mechanical testing of the specimens produced using the L-PBF technology was performed using an Instron 8802 testing machine (Instron, Norwood, MA, USA) at room temperature (20 °C). The tests were conducted on standard dog-bone specimens with a gauge length of 48 mm, a working section width of 3 mm, and a thickness of 2 or 3 mm. The working section was lightly polished to remove surface irregularities. Tensile tests were carried out in accordance with the ASTM E8/E8M [29] standard for metallic materials.

### 2.3. Multi-Objective Optimization—Grey Correlation Analysis

If the analysis of multi-objective DoE results shows high variability, meaning that the correlations between independent variables (process parameters) and dependent variables (material characteristics) are not statistically significant, and the trends in material characteristics are divergent, the application of the GRA method is advisable.

The GRA method involves the normalization of experimental data using Equation (2) for the “smaller-is-better” criterion and Equation (3) for the “larger-is-better” criterion. It also includes the calculation of the Grey Relation Coefficient (GRC) using Equation (4) and the determination of the Grey Relation Grade (GRG) using Equation (5).

Unlike DoE, which can only analyze and evaluate one material characteristic at a time, the GRA method allows for the simultaneous consideration of all material characteristics, including those with divergent criteria (“larger-is-better” and “smaller-is-better”).

(2) $$x_{ij}=\frac{\max_{j}y_{ij}-y_{ij}}{\max_{j}y_{ij}-\min_{j}y_{ij}},$$

(3) $$x_{ij}=\frac{y_{ij}-\min_{j}y_{ij}}{\max_{j}y_{ij}-\min_{j}y_{ij}},$$

where $y_{ij}$ is the value of the quality parameter *j* for the *i*-th combination of scanning parameters, and $y_{ij}=\max_{n}y_{ij}^{n}$—represents the most negative quality characteristic among the *n* combinations of scanning parameters.

(4) $$\xi_{ij}=\frac{\min_{i}\min_{j}|x_{j}^{0}-x_{ij}|+\zeta \max_{i}\max_{j}|x_{j}^{0}-x_{ij}|}{\left|x_{j}^{0}-x_{ij}|+\zeta \max_{i}\max_{j}|x_{j}^{0}-x_{ij}\right|},$$

where $x_{i}^{0}$ is the ideal normalized result (i.e., best normalized result) for the *j*-th quality characteristics, 0 ≤ *ζ* ≤ 1 is a distinguishing coefficient, the purpose of which is to weaken the effect of $\max_{i}\max_{j}|x_{j}^{0}-x_{ij}|$, when it becomes too big and thus enlarges the difference significance of the relational coefficient. It allows for adjusting the significance coefficients for each material characteristic, depending on their priority within the context of the study.

(5) $$\gamma_{i}=\frac{1}{m}\sum_{j=1}^{m}\xi_{ij},$$

where *m* is the number of material characteristics.

## 3. Results

### 3.1. Powder Characterization and Chemical Analysis

**Figure 1:**
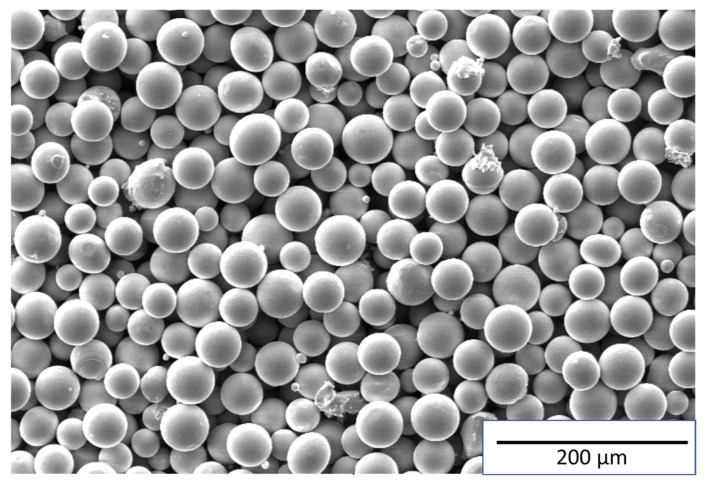
Particles of Ni-Cr-Mo-Nb based superalloy powder.

The spherical shape and narrow size distribution of metal powders are major requirements of AM for obtaining repeatable and reliable results [30]. Therefore, studies were conducted to analyze the morphological and granulometric composition of the Ni-Cr-Mo-Nb-based superalloy powder. The general appearance of the powder particles is shown in Figure 1.

Scanning electron microscopy revealed that most of the powder particles (95%) have a spherical shape, which is typical for powders produced using the plasma atomization process [31]. The particle size ranges from 15 to 53 µm. The oversize and undersize fractions account for 3.13% and 0.04%, respectively. The bulk density of the powder was 4.17 g/cm^3^, and its flowability was 20 s. The chemical composition of the powder is presented in Table 3.

### 3.2. Microstructure and Mechanical Properties

**Figure 2:**
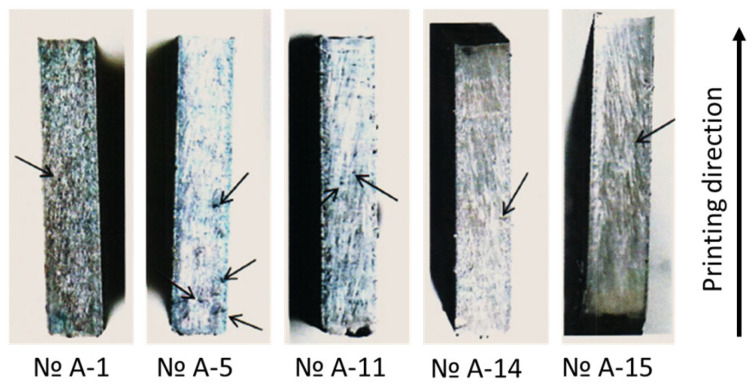
Macrostructure of the specimens. Arrows indicate gas pores and *LoF* defects.

Figure 2 shows the results of the macroanalysis of the specimens. It was found that all specimen surfaces contained gas pores with sizes up to 0.1 mm. The largest defects in the form of *LoF*, measuring up to 0.161 mm, were detected in specimens produced in Experiment No. 5.

**Figure 3:**
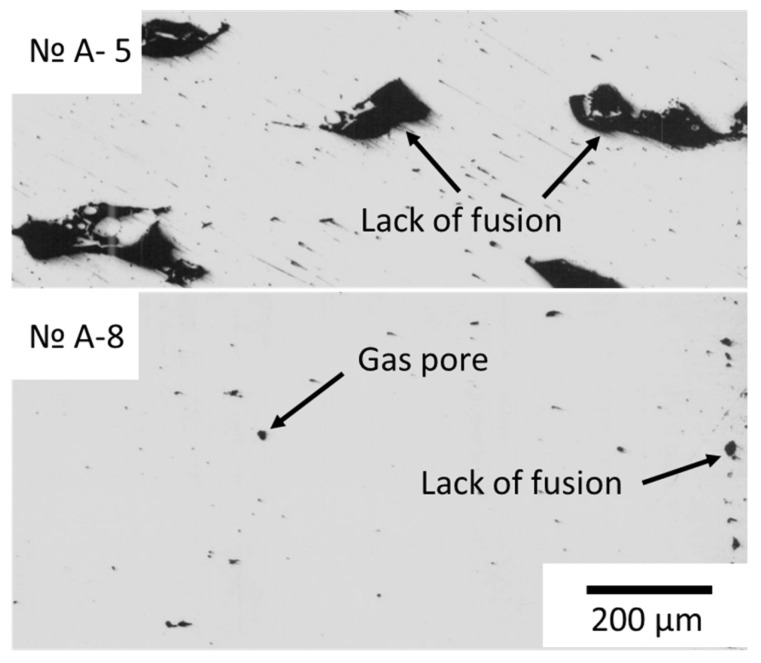
Defects in samples.

In the central region of all specimens, except those from Experiment A-5, isolated gas pores and LoF with a maximum size of 0.08 mm were observed. In the material of specimens produced in Experiment A-5, multiple *LoF* were observed (Figure 3).

**Figure 4:**
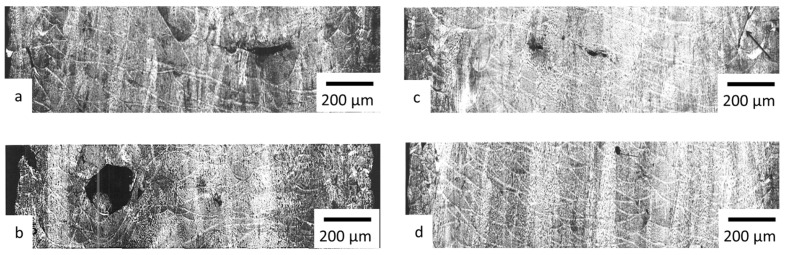
Optical micrographs of specimens processed at *E_V_* levels: (**a**) 69 J/mm^3^ (experiment number A-2); (**b**) 56 J/mm^3^ (experiment number A-5); (**c**) 76 J/mm^3^ (experiment number A-7); (**d**) 76 J/mm^3^ (experiment number A-15).

Adjusting laser power and scanning speed plays a key role in minimizing porosity [10]. Figure 4 shows cross-sections of specimens produced using various process parameters specified in the experimental design (Table 1).

Specimens produced with high volume energy density exhibit only a small number of fine pores, which is consistent with the results of other studies [13,16]. Notably, these specimens showed no cracks or significant *LoF* defects between layers. In contrast, specimens produced with low volume energy density values exhibited increased porosity as well as areas with *LoF* defects.

For further analysis and determination of correlations between process parameters and material characteristics, the microstructure desirability rank was assessed in accordance with Table 2.

### 3.3. Stage A: Optimization of L-PBF Parameters Using GRA

Table 4 presents the arithmetic mean values of the experimental results specified in the experimental design (Table 1).

To select an appropriate method for optimizing printing parameters, Pearson correlation coefficients were calculated (Table 5), characterizing the statistical significance of the relationships between factors (process parameters) and response variables (material characteristics).

The most significant correlation between the experimental responses was observed between yield strength (*σ*_0.2_) and ultimate tensile strength (*σ_β_*). The correlation coefficient of 0.59 indicates a moderately strong positive relationship. This is expected, as both parameters are related to the material’s microstructure (*Rank*), with correlation coefficients of 0.44 and 0.61, respectively.

An increase in the number of defects significantly reduces yield strength, which is consistent with the negative correlation value of −0.56 between yield strength (*σ*_0.2_) and the area of *LoF*.

An increase in laser power negatively affects the material’s mechanical properties (*σ*_0.2_ and *σ_β_*) within the current parameter range, as confirmed by correlation coefficients of −0.58 and −0.45, respectively. This may be due to defects caused by excessive laser energy, such as pore formation, overheating, or the “keyhole” effect [32].

Moreover, the correlation coefficients between the factors and the experimental responses are below 0.7 in all cases, indicating the complex nature of interactions and emphasizing the need for a comprehensive analysis. For further optimization of printing parameters, the Grey Relational Analysis (GRA) method was applied.

The analysis considered different degrees of desirability for the response characteristics: for material characteristics such as yield strength (*σ*_0.2_), ultimate tensile strength (*σ_β_*), elongation at break (*δ*), and microstructure desirability rank (*Rank*), the “larger-is-better” criterion was applied. In contrast, for parameters characterizing material defects, such as the area of *LoF* and Pores, the “smaller-is-better” criterion was used.

**Figure 5:**
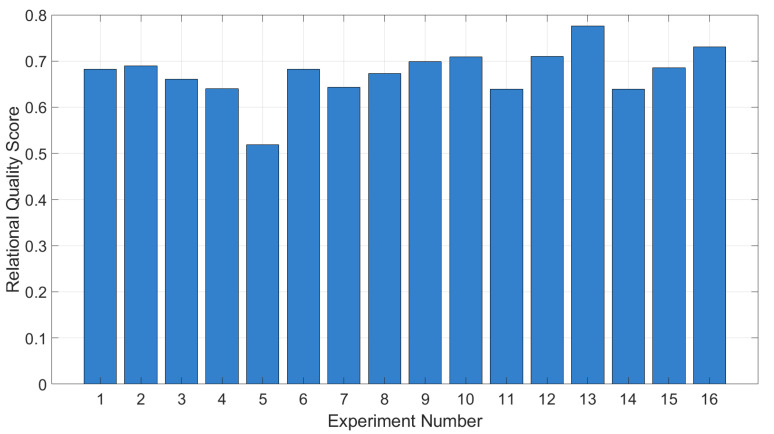
Experiment Stage A: The resulting relational quality scores.

The resulting relational quality assessments are presented in Figure 5. The higher the integrated relational assessment, the better the experimental result and the closer it is to the ideal normalized value of one. The calculated values show that the maximum integrated relational assessments are achieved in experiments A-13 and A-16, with values of 0.776 and 0.730, respectively.

### 3.4. Stage B: Optimization of L-PBF Parameters Using RSM

Based on the results of the Grey Relational Analysis (GRA) conducted in Stage A, printing parameters A-13 and A-16 were identified as having the highest integrated assessments of material characteristics. These parameters formed the basis for a new series of experiments with adapted printing parameters to refine the material characteristics (experiments B-1 to B-4).

To investigate the effect of sample thickness on material characteristics, additional experiments (B-5 to B-8) were conducted, replicating the parameters of experiments B-1 to B-4, except for the variation in sample thickness.

The experimental design for this stage was based on a fractional factorial design 2^4^//8. Table 6 presents the process parameters and the average values of the investigated material characteristics for each experimental point.

Given that the first series of experiments (Stage A) revealed a significant dependence of material characteristics on the thickness *S* of specimens subjected to tensile testing, a new process characteristic was introduced for a more accurate assessment of the experimental responses—the specific energy of layer fusion for a material sample with a width *S.* Numerically, this characteristic is equal to *E_V_×S×t*.

It is worth noting that the dependence of mechanical properties and microstructure on the thickness of the fused specimen is quite understandable, as the thermo-mechanical conditions of melting and solidification differ for specimens of varying thicknesses.

The results of the correlation analysis, demonstrating the degree of influence of individual process parameters on material characteristics, are presented in Table 7. High correlation coefficient values indicate the presence of statistically significant relationships between process parameters and material characteristics, confirming the possibility of constructing at least linear regression models for subsequent optimization using the gradient method.

To determine the most significant factors and obtain adequate regression dependencies of material characteristics on process parameters, linear models considering pairwise factor interactions of the form *Fi* (*a*, …, *b*, a*b) were analyzed, where *Fi* denotes the functional dependence of parameters *a*, …, *b* on specific material characteristics. The significance of the model coefficients was evaluated using Student’s *t*-test.

Based on the analysis, the following process parameters were found to have the most significant influence on material characteristics:

− σ_0.2_ = *F*_1_ (*E*_*V*_×*S*×*t*, *S*);
− σ_*B*_ = *F*_2_ (*E*_*V*_×*S*×*t*, *S*);
− *δ* = *F*_3_ (*P*, *t*);
− *LoF* = *F*_4_ (*E*_*V*_×*S*×*t*, *S*);
− *d* = *F*_5_ (*h*).

**Figure 6:**
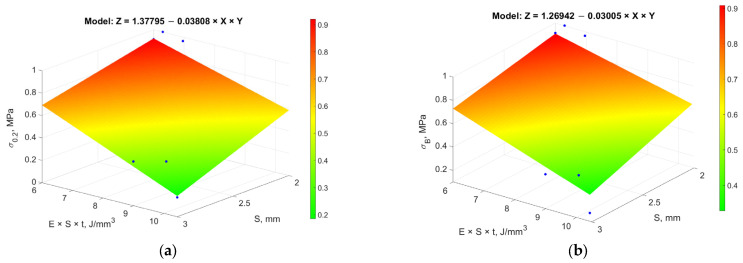

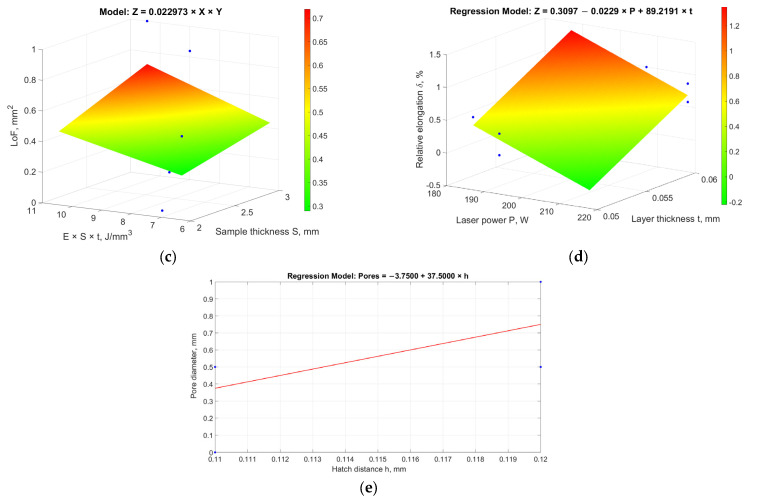
Stage B: Dependencies of material characteristics on process parameters: (**a**) dependence of yield strength (*σ*_0.2_) on sample thickness (*S*) and energy density (*E_V_×S×t*); (**b**) dependence of tensile strength (*σ_B_*) on sample thickness (*S*) and energy density (*E_V_×S×t*); (**c**) dependence of *LoF* area on sample thickness (*S*) and energy density (*E_V_×S×t*); (**d**) regression model showing the dependence of relative elongation (*δ*) on laser power (*P*) and layer thickness (*t*); (**e**) regression model showing the dependence of *d* on hatch distance (*h*).

The regression dependence for ultimate tensile strength (*σ_Β_*), obtained from data analysis with a determination coefficient *R* = 0.89, is presented in Figure 6a and expressed as follows:

(6) $$\sigma_{B}=\sigma_{0}+\alpha_{\sigma }E\cdot t\cdot S^{2}=\sigma_{0}+\alpha_{\sigma }\cdot P\cdot \frac{s}{V}\cdot \frac{s}{h},$$

where

*P*—laser power;
$\frac{s}{V}$—sample thickness normalized by scanning speed, characterizing the exposure time of the specimen to the melt front;
$\frac{s}{h}$—number of heat front passes within the layer, determining the heating non-uniformity in the layer perpendicular to the scanning speed.

Similar regression dependencies were obtained for the normalized yield strength (*σ*_0.2_) and the normalized maximum area of *LoF*, presented in Figure 6b,c, respectively, with determination coefficients *R* = 0.74.

The most adequate regression dependence for the normalized elongation at break (*δ*), presented in Figure 6d, shows that this material characteristic largely depends on the thickness of the tested specimen. It is expressed by the following equation:

(7) $$\delta =\delta_{0}+a_{\delta p}+\alpha_{\delta t}t$$

where

δ_0_—baseline elongation at break;
$a_{\delta p}\;and\;\alpha_{\delta t}$—coefficients determining the influence of the reduced laser power *P* and layer thickness *t*.

The gas pore diameter (*d*), according to the results of the correlation analysis (Table 7), mainly depends on the hatch distance (*h*) and is described by the linear relationship presented in Figure 6e:

(8) $$d=d_{0}+\alpha_{dh}h,$$

where

$d_{0}$—baseline gas pore diameter;
$\alpha_{dh}h$—coefficient determining the influence of hatch distance (*h*).

The dependencies presented in Figure 6 clearly show that the investigated range of process parameters is still significantly distant from achieving optimal material characteristics. This is confirmed by the substantial changes in the experimental responses when varying the process parameters, highlighting the need for further research to refine the range of process parameters that ensure optimal material characteristics.

In this regard, the optimization task was carried over to the next stage, Stage C, where it was addressed using an experimental-statistical approach, specifically applying the gradient ascent method. This method involves constructing a desirability function that assigns a “score vector” to a set of responses and selecting process parameters that maximize this score, ensuring convergence and stability of the optimization process.

### 3.5. Stage C: Optimization of L-PBF Parameters Using the Gradient Ascent Method

After the material characteristics deviating from optimal values were described by Equations (6)–(8), these equations were used to optimize the material characteristics, i.e., to move toward their optimal parameters. For this purpose, the Box–Wilson gradient ascent method [33] was applied.

In the first step, the step sizes for progressing toward the optimum were calculated for each factor (process parameter). The magnitude of the change in an independent factor (Δx) was proportional to the coefficient of that parameter in the linear regression equation. If a parameter (X) was part of multiplicative combinations with other parameters, its step size was calculated using a more complex approach. Therefore, at the initial stage, changes in the process parameters were calculated for the simplest dependencies.

Let us determine the step size *Δh* for the hatch distance (*h*) based on Equation (8) for the gas pore diameter. In accordance with the “smaller-is-better” principle, the step size *Δh* is calculated using the following expression:

(9) Δh=−0.375.

Based on Equation (7) for elongation at break (*δ*), the step size *ΔP* for laser power (*P*) was calculated. Considering the region of maximum elongation values on the response surface (Figure 6d) and following the “larger-is-better” principle, the step size *ΔP* was determined as follows:

(10) ΔP=−0.40175, t=1.

Let us introduce the desirability function Φ as a linear combination of normalized quality indicators multiplied by the corresponding significance coefficients:

(11) $$\Phi =k_{\sigma }{\bar{\sigma }}_{B}+k_{\delta }\bar{\delta }-k_{LoF}\bar{LoF}-k_{d}\bar{d},$$

where

$k_{i}$—significance coefficients for each quality characteristic;
${\bar{\sigma }}_{B},\bar{\delta },\bar{LoF},\bar{d}$—normalized values of the corresponding quality indicators.

Yield strength was not included in the desirability function because this material characteristic has a high degree of correlation with ultimate tensile strength (Table 7). The signs of the terms in the desirability function were determined according to the “larger-is-better” or “smaller-is-better” principles.

For further analysis, let us expand the desirability function for ultimate tensile strength σ_B_ into a Taylor series, assuming v=540 mm/s and t=0.6 mm. In this case, the increment ${\bar{\sigma }}_{B}$ is expressed as follows:

(12) $$\Delta {\bar{\sigma }}_{B}=a_{\sigma }\left({\left(\frac{s}{v}\right)}_{0}{\left(\frac{s}{h}\right)}_{0}\Delta p-2{\left(\frac{p}{v}\right)}_{0}{\left(\frac{s}{h}\right)}_{0}^{2}\Delta h\right),$$

where the subscript 0 indicates that the variable values preceding *Δp* and *Δh* correspond to the parameters of the optimal fusion mode (mode B-1, Table 6).

Similarly, for the *LoF* parameter:

(13) $$\Delta \bar{LoF}=a_{LoF}\left({\left(\frac{s}{v}\right)}_{0}{\left(\frac{s}{h}\right)}_{0}\Delta p-2{\left(\frac{p}{v}\right)}_{0}{\left(\frac{s}{h}\right)}_{0}^{2}\Delta h\right).$$

For the gas pore diameter:

(14) $$\Delta \bar{d}=a_{dh}\Delta h.$$

For elongation at break *δ* at *t* = 1, which corresponds to the region of maximum elongation values (Figure 6d):

(15) $$\Delta \bar{\delta }=a_{\delta p}\Delta p.$$

Then, the increment of the desirability function *ΔΦ* is determined by the following expression:

(16) $$\Delta \Phi =A_{p}\Delta p+A_{h}\Delta h,$$

where

(17) $$A_{p}=k_{\delta }a_{\delta }+\left(k_{\sigma }a_{\sigma }-k_{LoF}a_{LoF}\right){\left(\frac{s}{v}\right)}_{0}{\left(\frac{s}{h}\right)}_{0},$$

(18) $$A_{h}=-k_{d}a_{d}+2\left(k_{\sigma }a_{\sigma }-k_{LoF}a_{LoF}\right){\left(\frac{p}{v}\right)}_{0}{\left(\frac{s}{h}\right)}_{0}^{2}.$$

As a result, we obtain

(19) $$\Delta \Phi =A_{p}\Delta p+A_{h}\Delta h=\left(-k_{\delta }0.01297-\left(k_{\sigma }0.03005+k_{LoF}0.022973\right){\left(\frac{s}{v}\right)}_{0}{\left(\frac{s}{h}\right)}_{0}\right)\Delta p-\left(k_{d}37.5-2\left(k_{\sigma }0.03005+k_{LoF}0.022973\right){\left(\frac{p}{v}\right)}_{0}{\left(\frac{s}{h}\right)}_{0}^{2}\right)\Delta h.$$

The significance coefficients used in the desirability function are presented in Table 8.

In this case, the calculated increment coefficients of the desirability function for the point corresponding to the printing parameters of the best experiment (mode B-1, Table 6) are as follows:

$$A_{p}=-0.01235,\;A_{h}=-11.905.$$

For the second-best fusion mode in terms of material characteristics, with *t* = 0.05 (mode B-4, Table 6) the coefficients are as follows:

$$A_{p}=-0.00908,\;A_{h}=-37.5.$$

By taking the change in hatch distance (Δh) as the baseline value, the step size for changing the laser power $\left(\Delta p\right)$ for mode B-1 can be determined as follows:

$$\Delta p=\frac{A_{h}}{A_{p}}\Delta h=\frac{11.905}{0.01235}=963.8\Delta h\approx 1000\Delta h.$$

A similar calculation for mode B-4 gives:

$$\Delta p=\frac{A_{h}}{A_{p}}\Delta h=\frac{37.5}{0.00908}=4130.41\Delta h\approx 4000\Delta h.$$

Next, the gradient ascent was performed using the parameters Δh and Δp. Table 9 presents the gradient ascent steps for mode B-1, as well as the average response values from duplicate experiments at each step.

At the second step of the experiment (Table 9), the maximum mechanical properties of the material and minimal microstructural defects were achieved. At the third step, the optimization parameters began to decline, and this trend continued at the fourth step.

To determine the optimum within the current series of gradient ascent steps and to assess the feasibility of further iterations, GRA was applied.

As part of GRA, the desirability function coefficient (*ζ*) was set to 0.5 for all response characteristics, except for elongation at break (*δ*). For δ, the value of *ζ* was reduced to 0.35 due to the negative correlation of this parameter with yield strength (*σ*_0.2_) and ultimate tensile strength (*σ_β_)*, aiming to achieve optimal values for the other material characteristics.

**Figure 7:**
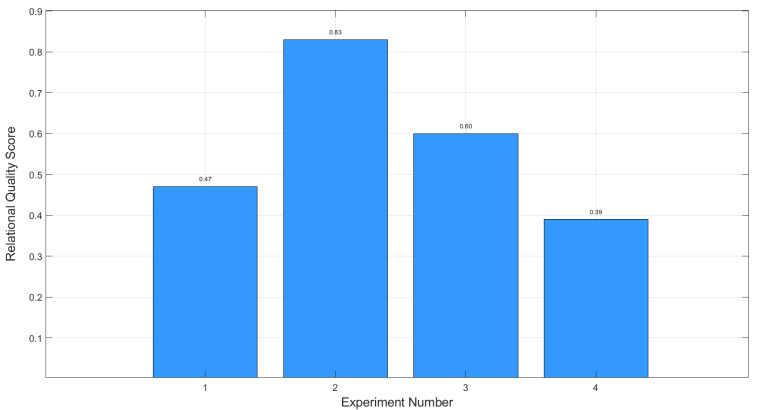
Stage C: The resulting relational quality scores.

The resulting relational quality assessments are presented in Figure 7. Calculations show that the maximum values of the integrated relational assessment are achieved in the second experiment (C-2), where the value reaches 0.83. This confirms the optimality of the process parameters used at this stage.

## 4. Discussion

At Stage A, the correlation coefficients between printing parameters and material characteristics were calculated and analyzed. The analysis revealed that the strongest correlation was observed between the material’s mechanical properties (*σ*_0.2_, *σ_B_*) and its microstructure (*Rank*), which is consistent with previous studies [10,13]. These studies demonstrated that microstructural defects significantly affect the material’s mechanical properties. The macrostructural analysis of the specimens (Figure 4) and the correlation matrix (Table 5) confirmed that volume energy density (*E_V_*) has the greatest influence on microstructure.

However, our data indicate that an increase in microstructure rank, which reflects a reduction in the number of defects and the presence of finer grains, leads to a decrease in the material’s overall plasticity. A previous study [34] showed that higher cooling rates and finer microstructures increase the material’s yield strength (*σ*_0.2_) but reduce elongation at break. This is due to the restricted movement of dislocations in fine-grained structures, which increases yield strength while decreasing the material’s ability to undergo plastic deformation.

Since the correlation coefficients were below 0.7, using traditional regression models proved challenging. Therefore, GRA was applied for multi-parameter optimization, considering conflicting optimization criteria (“larger is better” and “smaller is better”). The highest integrated quality scores were achieved with parameter sets A-13 (0.776) and A-16 (0.730), which were selected for further optimization in Stage B.

The application of RSM allowed for the development of regression models that describe the relationships between process parameters and material characteristics. Introducing the specific energy of layer fusion (*E_V_×S×t*) improved model accuracy. The analysis showed that σ_0.2_ and σ_β_ depend on *E_V_×S×t* and sample thickness (*S*), *δ* is influenced by laser power (*P*) and layer thickness (*t*), while gas pore diameter depends on hatch distance (*h*).

In a study conducted on Inconel 718 [35], the authors concluded that one of the optimal process parameter combinations lies within the power density range of 48 J/mm^3^. They reported mechanical properties comparable to those achieved in our study: *σ_β_*—1047 MPa, *σ*_0.2_—880 MPa, and elongation—37%. However, their methodology did not allow for the simultaneous optimization of defect levels. Specifically, defects such as lack of fusion (*LoF*) and gas pores remained notable, with the average size of lack of fusion defects being 0.217 mm and keyhole pores measuring 0.173 mm. This comparison illustrates a key difference between our approach and theirs, suggesting that our optimization method may be more effective in addressing the optimization of defects.

In contrast, at Stage C of our study, the Gradient Ascent Method was used to achieve the global optimum. The highest mechanical properties and the lowest defect levels were obtained at the second step (C-2): *σ*_0.2_—774.73 ± 4.94 MPa, *σ_β_*—1022.83 ± 5.19 MPa, elongation—23.1 ± 0.70%, *LoF* area—0.003 mm^2^, and gas pore diameter—0.02 mm. Further changes in parameters led to a decline in performance, confirming that the optimum was reached at C-2.

The correlation matrices (Table 4 and Table 7) clearly demonstrate the conflicting dependencies of material characteristics on process parameters. This creates the need to prioritize specific material characteristics depending on the task. In this study, the preferences for particular characteristics were defined using significance coefficients for the desirability function. Thus, the optimal process parameters can be calculated accordingly, based on the priorities established between minimizing defects and improving the mechanical properties of the material.

As noted in the Introduction, completely eliminating porosity in materials fabricated by L-PBF through process parameter optimization alone remains a challenging task. However, post-processing methods such as hot isostatic pressing (HIP) and the recently developed liquid-induced healing (LIH) have proven to be highly effective in mitigating residual defects [36,37]. These methods have demonstrated particularly high efficiency in healing small cracks and microstructural defects, which are often inevitable in L-PBF.

However, the presence of large internal defects and surface imperfections significantly reduces the effectiveness of these post-processing techniques. HIP is particularly limited in addressing surface-connected defects, as it primarily acts on enclosed voids under high pressure [38]. Similarly, the success of LIH depends on the ability of the liquid phase to fully penetrate and fill cracks, making it less effective for larger discontinuities.

The process optimization strategy demonstrated in this study allows for the production of a material with high mechanical properties and reduced microstructural defects, which can be further enhanced using these post-processing techniques. By minimizing the initial defect density, our approach improves the efficiency of HIP and LIH treatments, ensuring a more uniform and reliable final microstructure. This highlights the complementary nature of process optimization and post-processing in achieving high-performance materials for demanding engineering applications.

## 5. Conclusions

This study demonstrates the effectiveness of a three-stage multi-objective optimization approach for improving the mechanical properties and reducing defects in L-PBF-manufactured Ni-Cr-Mo-Nb superalloy components. Key findings and conclusions are as follows:

1. Multi-Objective Optimization Using GRA (Stage A): Due to the complex and multi-parameter nature of interactions between process parameters and material characteristics, Grey Relational Analysis was applied. This approach allowed for the simultaneous optimization of characteristics with conflicting criteria (“larger is better” and “smaller is better”). The highest relational quality scores were achieved with parameter sets A-13 (0.776) and A-16 (0.730).
2. Development of Regression Models Using RSM (Stage B): Response Surface Methodology enabled the development of regression models describing the relationships between process parameters and material characteristics. Introducing the specific energy of layer fusion (*E_V_×S×t*) as a key factor significantly improved the models’ accuracy. The analysis showed that *σ*_0.2_ and σ_β_ depend primarily on *E_V_×S×t* and sample thickness (*S*), while elongation at break (*δ*) is influenced by laser power (*P*) and layer thickness (*t*), and gas pore diameter depends on hatch distance (*h*).
3. Global Optimization Using the Gradient Ascent Method (Stage C): The Gradient Ascent Method was applied to identify the global optimum using a desirability function. The best results were obtained at the second ascent step (C-2), achieving a yield strength of 774.73 ± 4.94 MPa, tensile strength of 1022.83 ± 5.19 MPa, and elongation at break of 23.1 ±0.70%, with minimal *LoF* area (0.003 mm^2^) and gas pore diameter (0.02 mm). The subsequent steps led to a decrease in optimization performance, indicating that the global optimum was reached at C-2.

This multi-objective optimization framework provides a practical and effective methodology for improving the performance of L-PBF-manufactured components, contributing to the advancement of additive manufacturing technologies for high-performance applications.

## Figures and Tables

**Table 1:** Stage A: The experimental plan 4^4^ × 2^2^.

Experiment Number	*P*, W	*E_V_*, J/mm^3^	*V*, mm/s	*h*, mm	*t*, mm	*S*, mm
A-1	148	56	480	0.11	0.05	3
A-2	215	69	480	0.13	0.05	2
A-3	306	76	480	0.14	0.06	2
A-4	218	63	480	0.12	0.06	3
A-5	262	56	600	0.13	0.06	3
A-6	273	69	600	0.11	0.06	2
A-7	274	76	600	0.12	0.05	2
A-8	265	63	600	0.14	0.05	3
A-9	259	56	660	0.14	0.05	2
A-10	273	69	660	0.12	0.05	3
A-11	331	76	660	0.11	0.06	3
A-12	299	63	660	0.12	0.06	2
A-13	218	56	540	0.12	0.06	2
A-14	313	69	540	0.14	0.06	3
A-15	267	76	540	0.13	0.05	3
A-16	187	63	540	0.11	0.05	2

**Table 2:** Rank assessment of qualitative characteristics.

Characteristic	Quantitative/Qualitative Characteristic	Desirability Rank “More Is Better”
Grain structure type	Elongated grains	1–4
	Formation of “fish scale” structure (length-to-height ratio not exceeding 1.8)	4–8
Lack of interlayer bonding	Significant areas of non-penetration observed	1–2
	None	9–10
Pores	More than 3 within 500 mm^2^	6–8
	Less than 3 within 500 mm^2^	8–10

**Table 3:** Chemical composition of Ni-Cr-Mo-Nb superalloy powder.

Spectrum	Ni	C	Si	Mn	S	P	Cr	Mo	Nb	Al	Fe	N	O
wt.%	Base	0.041	0.73	0.24	0.005	0.004	27.1	7.2	3.1	1.53	1.95	0.2	0.017
vol.%	Base	0.143	2.469	0.262	0.019	0.017	29.7	5.52	2.85	4.465	1.95	1.261	0.094

**Table 4:** Stage A: Experimental results.

Experiment Number	Yield Stress *σ*_0.2_, MPa	Tensile Strength *σ_β_*, MPa	Relative Elongation *δ*, %	Rank	*LoF*, mm^2^	*d*, mm
A-1	858.08	1015.97	21.1	8	0.0189	0.08
A-2	719.81	1015.97	19.9	5	0.008	0.017
A-3	775.71	1000.28	16.2	8	0.008	0.04
A-4	710.00	991.45	24.3	5	0.015	0.05
A-5	667.83	943.40	20.1	5	0.161	0.04
A-6	768.84	1004.20	19.6	8	0.0024	0.04
A-7	744.32	1005.18	21.2	1	0.006	0.03
A-8	725.69	993.41	25.1	1	0.005	0.03
A-9	755.11	1030.68	20.8	5	0.0024	0.025
A-10	736.48	1004.20	23	5	0.0013	0.02
A-11	716.87	978.70	22.5	1	0.002	0.03
A-12	774.73	993.41	17.3	8	0.0015	0.02
A-13	803.16	1034.60	15.9	10	0.0002	0.02
A-14	719.81	961.05	19.6	1	0.0003	0.02
A-15	738.44	1004.20	23.1	1	0.0024	0.02
A-16	761.00	1060.10	19.85	7	0.001	0.03

**Table 5:** Stage A: Correlation matrix of process parameters and material characteristics.

Process Parameters/ Material Characteristics	*P*, W	*E_V_*, J/mm^3^	*V*, mm/c	*h*, mm	*t*, mm	*S*, mm	*σ*_0.2_, MPa	*σ_Β_*, MPa	*δ*, %	*Rank*	*LoF*, mm^2^	*d*, mm
*P*, W	1.00	0.58	0.54	0.35	0.44	0.15	−0.58	−0.45	−0.06	−0.44	−0.05	−0.50
*E_V_*, J/mm^3^	0.58	1.00	0	0.02	0	−0.17	−0.16	−0.23	0.12	−0.50	−0.37	−0.30
*V*, mm/c	0.54	0	1.00	−0.08	−0.03	−0.17	−0.17	−0.24	0.15	−0.23	0.03	−0.41
*h*, mm	0.35	0.02	−0.08	1.00	−0.06	−0.22	−0.29	−0.35	−0.02	−0.33	0.11	−0.35
*t*, mm	0.44	0	−0.03	−0.06	1.00	0.88	−0.52	−0.15	−0.45	0.27	0.24	0.03
*S*, mm	0.15	−0.17	−0.17	−0.22	0.88	1.00	−0.46	−0.15	−0.43	0.40	0.25	0.42
*σ*_0.2_, MPa	−0.58	−0.16	−0.17	−0.29	−0.52	−0.46	1.00	0.59	−0.18	0.44	−0.56	−0.05
*σ_Β_*, MPa	−0.45	−0.23	−0.24	−0.35	−0.15	−0.15	0.59	1.00	−0.41	0.61	−0.45	0.42
*δ*, %	−0.06	0.12	0.15	−0.02	−0.45	−0.43	−0.18	−0.41	1.00	−0.67	−0.02	0.14
*Rank*	−0.44	−0.50	−0.23	−0.33	0.27	0.40	0.44	0.61	−0.67	1.00	0.03	0.28
*LoF*, mm^2^	−0.05	−0.37	0.03	0.11	0.24	0.25	−0.56	−0.45	−0.02	0.03	1.00	0.25
*d*, mm	−0.50	−0.30	−0.41	−0.35	0.03	0.42	−0.05	0.42	0.14	0.28	0.25	1.00

**Table 6:** Stage B: The experimental plan 2^4^//8.

Experiment Number	*P*, W	*E_V_*, J/mm^3^	*V*, mm/c	*h*, mm	*t*, mm	*S*, mm	*E_V_×S×t*, J/mm	*σ*_0.2_, MPa	*σ_Β_*, MPa	*δ*, %	*LoF*, mm^2^	*d*, mm
B-1	218	56	540	0.12	0.06	2	6.72	727.65	1017.93	23.7	0.0156	0.03
B-2	207	58	540	0.11	0.06	2	6.96	750.21	1007.14	22.9	0.007	0.03
B-3	194	60	540	0.12	0.05	2	6.00	746.29	981.65	17.3	0.0055	0.03
B-4	187	63	540	0.11	0.05	2	6.30	753.15	1014.01	16.5	0.0238	0.03
B-5	218	56	540	0.12	0.06	3	10.08	710.98	987.53	21.3	0.0238	0.04
B-6	207	58	540	0.11	0.06	3	10.44	687.45	935.55	25	0.039	0.02
B-7	194	60	540	0.12	0.05	3	9.00	705.1	942.42	20.1	0.0336	0.04
B-8	187	63	540	0.11	0.05	3	9.45	673.72	893.39	21.7	0.02	0.03

**Table 7:** Stage B: Correlation matrix of process parameters and material characteristics.

Factors/ Experiment Response Characteristics	*σ*_0.2_, MPa	*σ_Β_*, MPa	*δ*, %	*LoF*, mm^2^	*d*, mm
*P*, W	0.03	0.43	0.64	−0.02	0.06
*E_V_*, J/mm^3^	−0.06	−0.41	−0.60	0.04	−0.10
*h*, mm	0.11	0.24	−0.17	−0.13	0.63
*t*, mm	−0.01	0.35	0.78	0.03	−0.21
*S*, mm	−0.89	−0.79	0.35	0.73	0.21
*E_V_×S×t*, J/mm	−0.87	−0.69	0.53	0.73	0.06
*σ*_0.2_, MPa	1.00	0.88	−0.51	−0.46	0.11
*σ_Β_*, MPa	0.88	1.00	−0.22	−0.62	0.07
*δ*, %	−0.51	−0.22	1.00	0.26	−0.35
*LoF*, mm^2^	−0.62	−0.46	0.26	1.00	−0.05
*d*, mm	0.07	0.11	−0.35	−0.05	1.00

**Table 8:** Stage C: Significance coefficients for the desirability function.

$k_{\sigma }$	$k_{\delta }$	$k_{LoF}$	$k_{d}$
1	0.7	1	1

**Table 9:** Stage C: Results of experiments using the gradient ascent method.

Step	*P*, W	*E_V_*, J/mm^3^	*V*, mm/c	*h*, mm	*t*, mm	*S*, mm	*Δh*	*ΔP*	*σ*_0.2_, MPa	*σ_Β_*, MPa	*δ*, %	*LoF*, mm^2^	*d*, mm
C-1	218	56	540	0.12	0.06	2	0	0	737.46	1014.01	22.4	0.0112	0.03
**C-2**	**207**	**58**	**540**	**0.11**	**0.06**	**2**	**−0.01**	**−10**	**774.73**	**1022.83**	**23.1**	**0.003**	**0.02**
C-3	194	60	540	0.10	0.05	2	−0.01	−10	731.58	1010.08	23.4	0.0117	0.02
C-4	187	63	540	0.09	0.05	3	−0.01	−10	730.60	991.45	23.1	0.014	0.03

## Data Availability

The original contributions presented in this study are included in the article. Further inquiries can be directed to the corresponding author.

## Nomenclature

Abbreviations:	
AM	Additive Manufacturing
L-PBF	Laser Powder Bed Fusion
SLM	Selective Laser Melting
DMLS	Direct Metal Laser Sintering
DoE	Design of Experiments
GRA	Grey Relational Analysis
RSM	Response Surface Methodology
HIP	Hot Isostatic Pressing
LIH	Liquid-Induced Healing
Symbols and Units:	
P (W)	Laser power
V (mm/s)	Scanning speed
h (mm)	Hatch distance
t (mm)	Layer thickness
E_V_ (J/mm^3^)	Volume energy density
S (mm)	Sample thickness
σ_0.2_ (MPa)	Yield strength
σ_B_ (MPa)	Ultimate tensile strength
δ (%)	Elongation at break
LoF (mm^2^)	Lack of fusion area
Rank	Microstructure desirability rank
d (mm)	Gas pore diameter
